# Coconut Oil Supplementation Does Not Affect Blood Pressure Variability and Oxidative Stress: A Placebo-Controlled Clinical Study in Stage-1 Hypertensive Patients

**DOI:** 10.3390/nu13030798

**Published:** 2021-02-28

**Authors:** Francisco A. O. Júnior, Clara R. Ruiz, Yohanna de Oliveira, Marco A. V. Barros, Alexandre S. Silva, Maria S. B. Santos, Vinícius J. B. Martins, Camille M. Balarini, Valdir A. Braga

**Affiliations:** 1 Health Sciences Center, Department of Physiology and Pathology, Federal University of Paraiba, Joao Pessoa 58051-900, Brazil; junior.ltf@gmail.com (F.A.O.J.); viniciusjbmartins@gmail.com (V.J.B.M.); camille.balarini@gmail.com (C.M.B.); 2 Biotechnology Center, Federal University of Paraiba, Joao Pessoa 58051-900, Brazil; clararittmeyer97@gmail.com; 3 Health Sciences Center, Department of Nutrition, Federal University of Paraiba, Joao Pessoa 58051-900, Brazil; yoh_0806@hotmail.com; 4 Medical Sciences Center, Federal University of Paraiba, Joao Pessoa 58051-900, Brazil; mavivo@cardiol.br; 5 Health Sciences Center, Department of Physical Education, Federal University of Paraiba, Joao Pessoa 58051-900, Brazil; alexandresergiosilva@yahoo.com.br (A.S.S.); sbrasileiro@yahoo.com.br (M.S.B.S.)

**Keywords:** nutritional approach, cardiovascular system, hypertension, coconut oil

## Abstract

Exploring an alternative to improve the clinical management of hypertension, we tested the hypothesis that food supplementation with coconut oil (EVCO), alone or combined with aerobic exercise training, could exert an antihypertensive effect (primary outcome) in patients with stage 1 hypertension. Forty-five hypertensive volunteers of both genders participated in a placebo-controlled clinical trial. The volunteers were submitted to 24-hour ambulatory blood pressure monitoring, analysis of blood pressure variability (BPV), measurement of serum malondialdehyde (MDA) and nutritional assessment. Results indicate that EVCO consumption had no adverse effects. The supplementation did not increase the caloric intake compared with placebo, and the dietary constituents were similar between groups, except for the saturated fats, especially lauric acid. The analysis of blood pressure indicated absence of antihypertensive effect of EVCO alone or combined with physical training. Furthermore, no effects on blood pressure variability and oxidative stress were observed in the supplemented hypertensive patients. Thus, despite the results observed in pre-clinical studies, the current clinical study did not provide evidence to support the use of coconut oil as an adjuvant in the management of hypertension in humans.

## 1. Introduction

The impact of hypertension on health continues to rise despite the advances in antihypertensive drug development by the pharmaceutical industry. In 2018, data from the American Heart Association indicated that 40.6% of deaths from cardiovascular disease in the U.S. were related to hypertension [1] and in the year 2035 the costs related to hypertension will exceed 220 billion dollars. Hypertension is a pandemic disease that affects approximately one-third of the world population [2].

Non-pharmacological approaches have been important adjuncts in the treatment of hypertension. Nutritional strategies such as using antioxidants and regular exercise have been shown to be beneficial [3,4,5], but more consistent clinical evidence is needed to strengthen the role of these alternatives in the management of hypertension. A recent strategy that has been used is the intake of coconut oil due to its nutritional, medicinal and cosmetic uses [6].

It has been demonstrated that coconut oil can increase high-density lipoprotein, improving the lipid profile [7,8], and increase main enzymes involved with the redox balance [8,9,10]. Despite these findings, currently there is controversy over the effects of coconut oil on human health [11,12]. In the cardiovascular system, preclinical studies of our group showed that coconut oil supplementation, alone or combined with exercise training, was able to induce antihypertensive response in spontaneously hypertensive rats [13]. In addition, it was shown that this effect was associated with an improvement in baroreflex response and attenuation of oxidative stress. Subsequently, it was found that lauric acid, the main fatty acid isolated from coconut oil, was able to induce a dose-dependent reduction in blood pressure (BP), as well as to promote vasorelaxation of isolated rat superior mesenteric artery [14].

However, there are no reports on the effects of coconut oil supplementation in relation to the cardiovascular system in humans. Studies that focus on the relationship of coconut oil consumption with physiological variables of interest such as BP and BP variability can elucidate the potential of coconut oil as an adjuvant alternative in the clinical management of hypertension. Thus, we tested the hypothesis that coconut oil supplementation, isolated or combined with physical training, could exert antihypertensive effects in patients with stage 1 hypertension.

## 2. Materials and Methods

### 2.1. Subjects and Experimental Groups

Hypertensive patients were recruited to participate in this placebo-controlled study. Patients with stage 1 systemic arterial hypertension (mild hypertension) were included. This degree of hypertension is characterized by levels of systolic blood pressure between 140 and 159 mmHg and/or diastolic blood pressure between 90 and 99 mmHg [15]. Patients were evaluated by 24-hour blood pressure monitoring to confirm the diagnosis of hypertension and for follow-up during the experimental period. The study included patients of both genders with a minimum age of 20 and a maximum age of 64 years. Patients with diabetes mellitus or under pharmacological treatment for hypertension were not included. The study was conducted in accordance with the ethical guidelines of the Declaration of Helsinki and approved by the Research Ethics Committee of the University Hospital of the Federal University of Paraiba, Brazil (protocol nº 1.523.128/2016). Written informed consent was obtained from all volunteers prior to participation. In addition, this clinical protocol was approved in the Brazilian Registry of Clinical Trials (nº RBR-5s9bhc).

**Figure 1:**
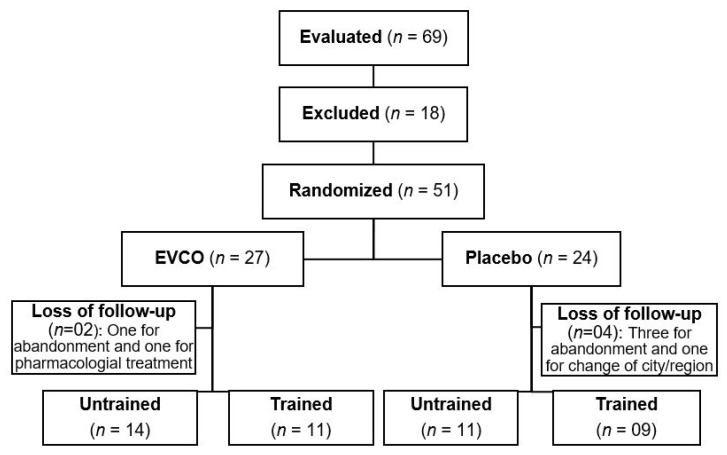
Flowchart of the study design.

The sample size was based on the outcome of reduced systolic blood pressure obtained by Perona et al. [16], considering a mean difference of 14 mmHg with a standard deviation of 10 and 8 mmHg for each group. The sample size was calculated with the open license tool at (https://www.openepi.com (accessed on 1 February, 2017)) and, as a result, a minimum of seven subjects for each group was obtained, considering a confidence interval of 95% and a statistical power of 80%. Our study included 51 hypertensive patients who were allocated by simple random distribution (1:1) using a freely available website (www.randomizer.org (accessed on 1 February, 2017)). These patients were followed up in the experimental groups: group supplemented with extra-virgin coconut oil—EVCO; group supplemented with placebo; group supplemented with EVCO plus physical training (EVCO-training); and group supplemented with placebo plus physical training (placebo-training). Six patients did not complete the treatment protocol and were excluded. Two of them were from the EVCO group (one on a physical training protocol and one untrained). The patient in the training group dropped out of the study, and the other patient started pharmacological treatment by decision of the cardiologist on the study team. The remaining four losses were from patients supplemented with the placebo intervention (one from the trained group and three from the untrained group). The patient in the trained group had to move from the study city, and the other three dropped out the study. Considering blood pressure analyzes, two records were excluded (one from the placebo group and one from the EVCO group) because they did not reach more than 80% of the valid measures after 24-hour monitoring. Thus, we had the following experimental groups: EVCO, *n* = 14; Placebo, *n* = 11; EVCO-training, *n* = 11; and Placebo-training, *n* = 9, Figure 1.

### 2.2. Study Design and Experimental Protocols

This is a randomized, single-blinded, placebo controlled clinical trial. Patients were initially submitted to 24-hour ambulatory blood pressure monitoring according to VI Brazilian guidelines for hypertension [15]. Blood collection was performed to biochemical analysis. After the initial evaluation, the patients in each group were submitted to food supplementation with EVCO capsules or placebo for a period of 30 days. Twenty-four hours after the last day of this intervention period, patients were again evaluated for initial variable.

The patients receiving EVCO or placebo were instructed to intake 10 capsules a day within the main meals as follows: three during the breakfast, four during lunch and three during dinner. The EVCO groups received 10 mL/day (1 mL in each capsule) of EVCO (*Cocos nucifera* L.) [17]. Both preparations were standardized in a specialized pharmacy (registration with the Ministry of Health Nº. 5.6372.0016.001-4) (Table 1).

The training protocol consisted of four weeks of aerobic training (walking and/or running on an ergometric treadmill), three times per week, lasting 60 min, of moderate intensity (50% to 70% heart rate reserve) [18]. A one mile field test was performed prior to the protocol to estimate the maximum oxygen consumption and to obtain the maximum heart rate [19]. The heart rate of training was determined based on the reserve heart rate [20], monitored by Polar^®^ frequency meter model M200 (Polar Electro, Kempele, Finland), and supervised by rating of perceived exertion [21].

### 2.3. Nutritional Evaluation

The dietary intake of each patient was assessed by a three day food recorder, two representing the weekday diet and one representing the weekend diet. The average food consumption over the three days was used for nutritional assessment. The analysis was performed by a nutrition professional using a nutritional software (Dietwin Plus^®^, version 3048/16, Porto Alegre, Brazil). The nutritional parameters of total energy intake, carbohydrates, total proteins and fats, saturated fatty acids (including lauric acid), unsaturated fatty acids, intake of sodium and antioxidants were selected for analysis.

### 2.4. Thiobarbituric Acid Reactive Substances (TBARS) Assay and Clinical Biochemistry Measurements

To evaluate lipid peroxidation, serum samples were collected to measure levels of malondialdehyde (MDA) determined by TBARS assay [22]. After blood collections, the samples were centrifuged at 5000× *g* for 10 min to obtain serum. Then, 250 µL of serum were stored at 37 °C for 1 h, after which 400 µL of 35% perchloric acid was added, and the mixture was centrifuged (14,000× *g*/4 °C) for 20 min. Four hundred and fifty microliters of supernatant was removed and mixed with 0.6% thiobarbituric acid and incubated at 90 °C for 1 h. Then, absorbance at 532 nm was measured. A standard curve was generated using 1,1,3,3-tetrametoxypropane. Results were expressed as nmol/mL.

Serum was used to determine total cholesterol, triglycerides, low density lipoproteins (LDL-cholesterol), high density lipoproteins (HDL-cholesterol) and glucose using colorimetric standardized commercial kits (Blioclin-Quibasa, MG, Brazil). Non-HDL-cholesterol is calculated by subtracting HDL-cholesterol from total cholesterol.

### 2.5. Ambulatory Blood Pressure Monitoring [ABPM) for 24 Hours and Anthropometric Measures

The BP was measured for a period of 24 h using a Dyna MAPA + *Cardios*^®^ device (São Paulo, Brazil). The equipment was programmed to perform measurements every 15 min during the waking period and every 30 min during sleep, according to the recommendations of the 4th Guidelines for Ambulatory Blood Pressure Monitoring and 2nd Guidelines for Residential Blood Pressure Monitoring [23]. The data obtained during registration were used to determine the mean values of systolic diastolic blood pressure and mean. In addition, they were also used to establish the parameters of blood pressure variability, standard deviation and average real variability [24]. In addition, patients were weighed and measured using a portable stadiometer (1 mm accuracy) and a body weight apparatus (OMRON^®^ model HBF-514C, Shiokoji Horikawa, Shimogyo-ku, Kyoto, Japan). Body mass index was calculated using the formula weight/(height)^2^.

### 2.6. Statistical Analysis

The analysis of normality was performed by Shapiro-Wilk test. Non-normal variable (malondialdehyde) was log-transformed from their original values. In the nutritional analysis, a comparison between means of the diet components the placebo and EVCO periods was made by unpaired *t* Student test. For the other analyses, a one-way ANCOVA corrected by age was performed with repeated measurements, followed by Bonferroni post-test. The data were expressed as means and standard deviation and the significance level was adopted with *p* < 0.05. The effect size values were expressed by the partial *eta* square (*η*^2^).

## 3. Results

### 3.1. Baseline Profile of Participants

Table 2 displays the baseline assessment of the patients treated in this study. The patients showed body mass index (BMI) in the overweight range and total cholesterol, triglycerides and non-HDL cholesterol with borderline concentrations.

### 3.2. Nutritional Analysis of Food Records

The records obtained from the food diaries of the patients supplemented with coconut oil or placebo indicates that there was no difference in the energy intake (1961 (1700–2222) vs. 1678 (1450–1905) kcal; *p* = 0.134), carbohydrate intake (252.8 (200.1–305.5) vs. 213.2 (177.9–248.4) g; *p* = 0.278) and total fats intake (69.1 (56.7–81.5) vs. 55.7 (40.5–70.8) g; *p* = 0.161). However, considering the fractions of saturated fats (29.3 (23.5–35.1) vs. 18.5 (12.0–24.8) g; *p* = 0.017) and the amount of lauric acid (4.64 (4.50–4.77) vs. 0.20 (0.07–0.34) g; *p*<0.001) ingested in periods of coconut oil and placebo supplementation, respectively, a greater amount of saturated fats and lauric acid is observed for patients supplemented with coconut oil. For the other dietary constituents evaluated (proteins, cholesterol, linolenic acid, trans fats, sodium, zinc, vitamins B12, B9, A, C, E and β-carotene) there was no significant difference between the periods of supplementation with EVCO and placebo (data not shown). In addition, no adverse effects (diarrhea, constipation, nausea, colic, migraine, among others) were observed or reported in relation to the use of 10 mL/day of EVCO during the 30-day period, suggesting that this pattern of dietary supplementation is fully safe in humans.

### 3.3. Analysis of 24-Hour Blood Pressure Monitoring

**Figure 2:**
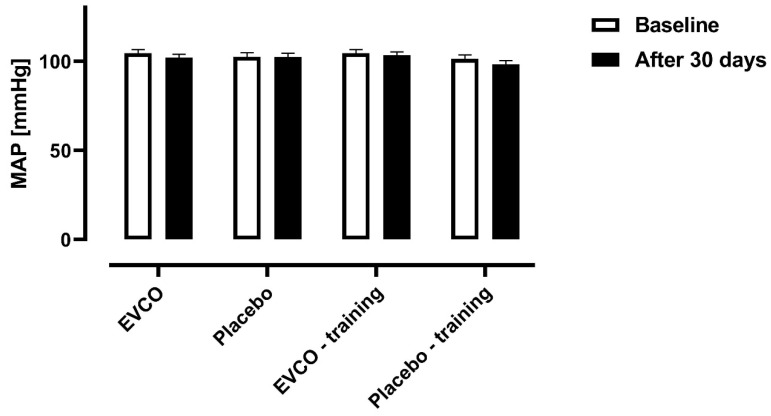
Comparison of mean blood pressure levels between experimental groups. Data expressed as means and SD (*n* = 43). Mean Arterial Pressure (MAP). One-way ANCOVA of repeated measures corrected for age: Time factor (*p* = 0.119 and *η*^2^ = 0.063) and Group factor (*p* = 0.482 and *η*^2^ = 0.062). There were no significant interaction of effects (*p* = 0.446 and *η*^2^ = 0.067).

Figure 2 shows the mean blood pressure values obtained from the 24-hour blood pressure record of hypertensive patients, before and after the 30-day intervention for all experimental groups. No differences were found.

### 3.4. Analysis of Serum Concentrations of Malondialdehyde

**Figure 3:**
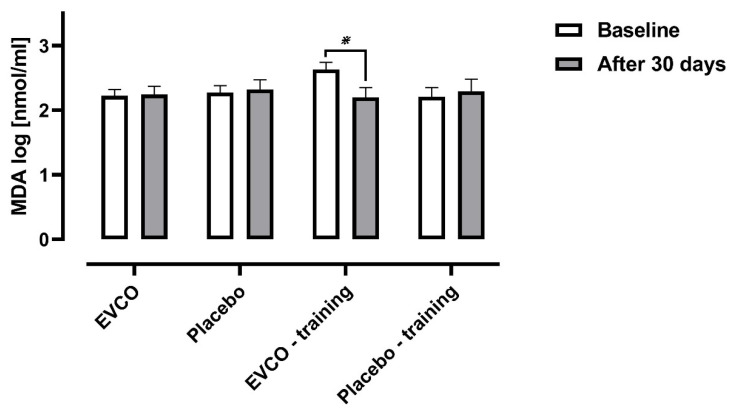
Comparison of serum malondialdehyde concentrations in hypertensive patients after 30 days of supplementation with EVCO or placebo, combined or not with aerobic physical training. One-way ANCOVA of repeated measures corrected for age, followed by a Bonferroni *post-test* (*n* = 45): time factor (*p* = 0.559 and *η*^2^ = 0.009); group factor (*p* = 0.644 and *η*^2^ = 0.04) and interaction factor (*p* = 0.015 and *η*^2^ = 0.227). * Post hoc for interaction: *p* = 0.001 (EVCO-training group).

There is no EVCO-induced effect on serum MDA concentrations (nmol/mL) assessed after the experimental intervention compared to control intervention (EVCO (2.22 ± 0.10 vs. 2.24 ± 0.13); Placebo (2.27 ± 0.11 vs. 2.32 ± 0.15); EVCO *plus* training (2. 63 ± 0.10 vs. 2.20 ± 0.14); and Placebo *plus* training (2.21 ± 0.12 vs. 2.29 ± 0.17); *p* = 0.644 and *η*^2^ = 0.009). However, there is an interaction effect between time and group that appears to be promoted as a function of a decrease between initial and final MDA concentrations among patients who were supplemented with EVCO and trained (*p* = 0.015 and *η*^2^ = 0.227) (Figure 3).

### 3.5. Analysis of Blood Pressure Variability

**Figure 4:**
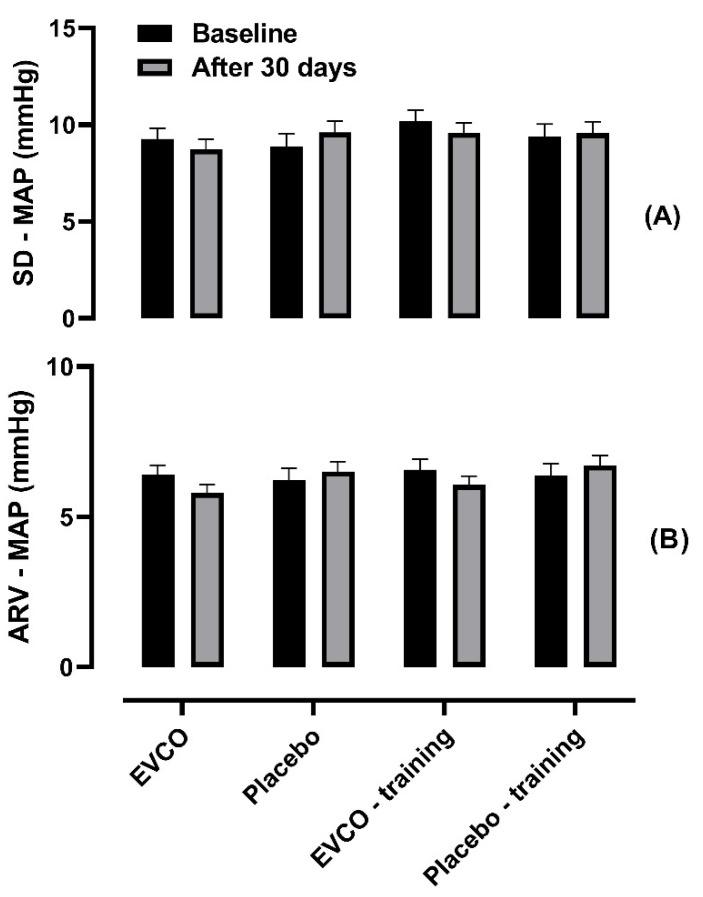
Comparison of the blood pressure variability in hypertensive patients after 30 days of supplementation with EVCO or placebo, combined or not with aerobic physical training. One-way ANCOVA of repeated measures corrected for age (*n* = 43). (**A**) SD–MAP: (Time factor (*p* = 0.696 and *η*^2^ 0.004); group factor (*p* = 0.595 and *η*^2^ = 0.048); and interaction factor (*p* = 0.233 and *η*^2^ = 0.105). (**B**) ARV–MAP: (Time factor (*p* = 0.848 and *η*^2^ 0.001); group factor (*p* = 0.673 and *η*^2^ = 0.039); and interaction factor (*p* = 0.434 and *η*^2^ = 0.069).

The analysis of blood pressure variability data, represented by standard deviation (SD) and average real variability (ARV), showed that supplementation with EVCO was not effective within the assessed groups: SD–MAP: (EVCO (9.27 ± 0.54 vs. 8.73 ± 0.510); Placebo (8.90 ± 0.65 vs. 9.60 ± 0.60); EVCO *plus* training (10.2 ± 0.57 vs. 9.58 ± 0.53) and Placebo *plus* training (9.40 ± 0.64 vs. 9.56 ± 0.60)); and ARV–MAP: (EVCO (6.39 ± 0.33 vs. 5.80 ± 0.28; Placebo (6.22 ± 0.40 vs. 6.50 ± 0.34); EVCO *plus* training (6.57 ± 0.34 vs. 6.07 ± 0.29); and Placebo *plus* training (6.37 ± 0.40 vs. 6.71 ± 0.33)) (Figure 4).

## 4. Discussion

Original preclinical studies provided evidence that both *in natura* coconut oil and its main isolated constituent, lauric acid, were able to evoke antihypertensive effect in spontaneously hypertensive rats and vasorelaxant effect in superior mesenteric artery, respectively [13,14]. It was also shown that these effects could be related to a reduction in oxidative stress in these experimental models. However, from a translational perspective, no study has been conducted to assess the possible effects of coconut oil in humans with hypertension.

Clinical studies with specific subpopulations and testing non-pharmacological alternatives are essential. Thus, considering the preliminary research, this was the first trial designed to assess the effects of EVCO in patients with systemic arterial hypertension. So, this study represents an effort to test an unknown outcome on the use of coconut oil in humans. In addition, we highlight the fact that the present study controlled precisely (through supplementation with capsules) the amount of EVCO effectively ingested. An important proportion of the studies that focus on this natural product did not properly control the amount of EVCO ingested; instead, it was included in meal preparation, without precise delimitations of its consumption [25,26]. Our study was placebo-controlled and used a biologically inert substance as a control intervention, the pharmaceutical starch. This is relevant because several studies have compared the use of coconut oil with other vegetable oils, which sometimes also have biological effects, causing complications in the interpretation of the results obtained [27,28].

Regarding the primarily investigated outcome, there was no antihypertensive effect associated with supplementation with EVCO in patients with stage 1 arterial hypertension tested in this study, unlike what was evidenced in the preclinical study with an animal model of essential hypertension [13]. Clinically, it should be noted that the diagnostic parameter used in this study was consistent, since a 24-hour blood pressure monitoring technique was used. The ABPM is fundamental in the evaluation of the efficacy of antihypertensive drugs in clinical trials and, consequently, very useful in the clinical management of hypertension [29]. This type of cardiological evaluation has a high diagnostic power [30], as it eliminates stress-induced momentary hypertension, called white coat hypertension. Sometimes, an isolated outpatient measure may not reflect the patient’s real blood pressure [31]. In some cases, a sympathetic discharge associated with the situation of being evaluated by a cardiologist is sufficient to induce a pressure response [32]. This evidence reinforces caution in issuing false-positive results. Finally, this monitoring reflects the pressure behavior during the entire 24 h period. On average, approximately 70 (seventy) blood pressure measurements are performed, without interference from the human factor during the checks, minimizing the measurement bias.

With regards to the difference in response evoked by coconut oil supplementation between pre-clinical and clinical studies, it is prudent to consider the distinctions inherent to each experimental model that was used in each study. The two studies involve specimens with different structure and metabolism and also with different experimental conditions. In the preclinical study, the dose of coconut oil administered was 2 mL per day for animals weighing between 200–300 g. Based on this dosage, if this was extrapolated for use in adult humans (average weight 70 Kg), an amount that would exceed 500 mL of EVCO per day would be necessary [33]. The dose used in this study (10 mL/day) was based on the study by [17] which proved to be safe, with no adverse effects recorded. [34] demonstrated that virgin coconut oil (1.4 mL/kg weight) prevents blood pressure elevation in rats fed palm oil. The experiment used 32 male Sprague-Dawley rats and measurements were performed every four weeks for a total period of 16 weeks by tail-cuff method. It is not clear that the beneficial effects attributed to coconut oil obtained in animal studies can be extended to humans. For example, Khaw et al. [28] compared three types of diets: EVCO, extravirgin olive oil and unsalted butter. The participants incorporated 50 g of the respective fats into their usual diet for a period of four weeks. These authors did not report any difference in pressure levels after the experimental period. On the other hand, Perona et al. [16] observed a reduction in systolic blood pressure levels in elderly hypertensive patients after using 60 g/day virgin olive oil for four weeks. Indeed, unlike what is observed in relation to coconut oil, Massaro et al. [35], through a distinguished review work, showed that there is relative similarity of effects between pre-clinical and clinical studies addressing the use of olive oil to treat hypertension. Summarizing, Wallace [25] performed a systematic review on the effects of coconut oil and concluded that the available evidence is sufficient only to encourage new studies with more methodological robustness. Another important aspect that deserves consideration is that, in order to avoid the confounding effects of medication, we opted for stage 1 hypertensive patients. Whether coconut oil may exert some beneficial effect on blood pressure of patients with higher blood pressure levels remains to be elucidated.

Although no effect on blood pressure was observed, a possible effect of EVCO on the autonomic function of patients was investigated. Two main factors motivated this experimental approach. First, in the pre-clinical study it was seen that, besides the antihypertensive activity, the supplementation with EVCO was associated with a reduction in oxidative stress and with an improvement in the baroreflex response in the treated animals [13]. Second, this interrelationship between a disturbance of blood pressure control mechanisms and the development of hypertension is widely reported [36]. A reduction in the effectiveness of control mechanisms can be expressed by increases in blood pressure oscillations over time [37]. It is important to emphasize that the autonomic function can also be explored through the analysis of blood pressure variability. This alternative constitutes a non-invasive tool capable of providing parameters related to autonomic activity based on blood pressure oscillations over time [38].

Blood pressure variability is not a physiological variable routinely accessed in clinical practice, although according to Hsu et al. [39], it is recognized as a prognostic factor independent of blood pressure level [24]. Evidence shows that the adverse cardiovascular consequences of high blood pressure may be the result of increased variability and not high blood pressure alone [40,41]. In addition, there is a strong association between increased BP variability and subclinical progression of target organ damage [42]. Two indexes of BP variability were used, the standard deviation and the average real variability. They are not redundant analyses, because according to Mena [24], ARV offers an additional reinforcement in the variability representation. Our results indicated no effect of ECVO supplementation on the variability indices evaluated. Furthermore, no effect interaction between the groups was observed, although a moderate effect size persists considering the average real variability. The analysis indicates that physical training did not prove to be determinant for this response. Although aerobic exercise is a useful complement to control hypertension, it does not necessarily affect blood pressure variability [43]. In addition, the most effective training protocols for lowering blood pressure levels are longer (minimum 8–12 weeks) [44]. The associative effect, i.e., the potentiating effect promoted by the supplementation with EVCO in combination with training, seen in the pre-clinical study [13], has not been fully reproduced in humans.

In addition, through the antioxidant potential of several coconut oil constituents [45,46] and the relationship between reactive oxygen species and hypertension control mechanisms [47,48] analyses were conducted to estimate the serum oxidative stress of hypertensive patients. This effect was evaluated by comparing the serum concentrations of MDA, a byproduct of lipid peroxidation, in different experimental conditions. As previously mentioned, this interaction between oxidative stress and damage to the autonomic function is well documented [49,50] and increase in the variability has been linked to alterations in autonomic function, including sympathetic hyperactivity [51]. Following the findings of ineffectiveness obtained on the cardiovascular variables of blood pressure and blood pressure variability, it was found that nutritional supplementation with EVCO was not able to interfere with the oxidative stress accessed through the MDA. Only an isolated effect of the interaction was seen in the group supplemented with EVCO which also participated in the physical training program. Although this effect reproduces, at least in part, the possible amplifying response of EVCO combined with exercise training, it was obtained in a group that started from a higher baseline value of MDA.

In fact, the effectiveness of this antioxidant property inherent to EVCO has been attributed almost exclusively to animal models. Nevin and Ramajohan [9] showed that virgin coconut oil was related to an increase in the activity of the enzymes glutathione peroxidase, catalase and superoxide dismutase in organs such as liver, heart and kidneys. Famurewa et al. [8] demonstrated that this increase in the activity of hepatic catalase and superoxide dismutase (but not GPx) occurred in a concentration dependent on EVCO and that they were related to a decrease in MDA. This response was also observed in homogenate of rat heart muscle [10]. Studies in humans focusing on the antioxidant property of coconut oil are rare and less consistent. Sabitha et al. [52], for example, found no significant differences between individuals treated with coconut or sunflower oil in relation to oxidative stress. Additionally, the results indicated that physical exercise was not able to promote significant changes in oxidative stress after 30 days. It seems that this relationship between exercise and oxidative stress is complicated and can be modulated in several levels of intensity, type and duration of training [53,54]. Regarding other possible sources of exogenous antioxidants offered by the diet during the experimental period that could eventually have interfered with the responses discussed in this study, it should be noted that the nutritional follow-up implemented represented an additional factor to reinforce the evidence presented.

In conclusion, the supplementation with EVCO did not have an antihypertensive effect in patients with stage 1 hypertension. It also showed no effectiveness on blood pressure variability and oxidative stress levels in humans. Thus, although coconut oil supplementation (10 mL/day) was shown to be safe as a nutritional approach and without significant impacts on the caloric load intake, it was not able to reproduce the effects obtained in an experimental model of hypertension when translated to hypertensive patients. Finally, it should be ratified that complementary studies are necessary to determine if doses different from the current trial can evoke additional or diverse effects.

## Figures and Tables

**Table 1:** Nutritional information on coconut oil supplementation.

Nutritional Constituents	Quantity	Dv % *
Total calories (Kcal)	90	5
Total fats (g)	10	20
Saturated fats (g)	7.5	35
Monounsaturated fats (g)	2.5	**
Lauric acid (g)	5	**
Myristic acid (g)	2.5	**
Oleic acid (g)	2.5	**

Equivalent to the content of 10 capsules with extra virgin coconut oil. * Percentages of daily values in relation to a 2000 kcal diet. ** Daily values not established.

**Table 2:** Baseline clinical characteristics.

Baseline Parameters	EVCO (*n* = 14)	Placebo (*n* = 11)	EVCO *plus* Training (*n* = 11)	Placebo *plus* Training (*n* = 9)	*p* Valor
Men *n* (%) †	7 (46.0%)	7 (63.0%)	8 (73%)	7 (88%)	0.390
Age (years)	49.6 ± 9.9	35.0 ± 9.3	42.8 ± 9.0	37.0 ± 12.3	0.006 #
BMI	29.8 ± 5.8	28.6 ± 5.4	27.9 ± 4.7	31.4 ± 4.2	0.622
Total cholesterol	198.6 ± 36.3	163.2 ± 36.3	195.0 ± 48.5	197.1 ± 85.5	0.433
HDL-cholesterol	38.9 ± 12.7	45.7 ± 13.0	55.2 ± 10.3	38.4 ± 12.9	0.009#
LDL-cholesterol	82.2 ± 8.8	60.7 ± 16.1	94.6 ± 26.2	77.2 ± 27.9	0.039#
Non-HDL cholesterol	167.3 ± 59.3	117.5 ± 34.9	139.7 ± 50.1	158.6 ± 84.4	0.355
Triglycerides	158.6 ± 66.2	108.6 ± 47.0	168.1 ± 51.3	148.4 ± 97.2	0.299
Glucose	89.2 ± 13.1	82.2 ± 12.8	90.9 ± 10.0	89.5 ± 14.4	0.492
MDA ^a^	2.29 ± 0.3	2.18 ± 0.2	2.65 ± 0.3	2.16 ± 0.2	0.012 #
SAP ^b^	134.6 ± 8.2	131.3 ± 9.1	134.2 ± 8.6	132.1 ± 6.2	0.303
DAP ^c^	87.7 ± 8.1	88.8 ± 9.9	89.9 ± 6.6	88.0 ± 4.4	0.898
MAP ^d^	103.2 ± 7.2	102.7 ± 9.0	104.5 ± 6.2	102.5 ± 3.3	0.765
SD-MAP ^e^	9.3 ± 1.6	8.9 ± 1.9	10.2 ± 2.2	9.3 ± 1.9	0.552
ARV-MAP ^f^	6.5 ± 1.3	6.1 ± 0.9	6.5 ± 1.3	6.4 ± 0.7	0.898
VO_2_max ^g^	-	-	43.4 ± 5.7	39.8 ± 4.6	0.140

(HDL) = High Density Lipoprotein; (LDL) = Low-density lipoprotein; others lipidic profile parameters and glucose expressed in (mg/dL). BMI = Body Mass Index (kg/m^2^). ^a^ The values of malondialdehyde (MDA) are expressed in log (nmol/mL); ^b^, ^c^ and ^d^ Mean values of systolic (SAP), diastolic (DAP) and mean blood pressure (MAP) (mmHg) obtained in awake patients using 24-hour blood pressure monitoring, respectively; and ^e^ Standard deviation (SD) and ^f^ Average real variability (AVR) of the mean arterial pressure (mmHg); ^g^ (VO_2_max) = Maximum oxygen consumption (mL·kg^−1^·min^−1^) estimated by 1-mile field test for the patients in the training protocol. Data expressed as mean ± standard deviation or n(%); † Person Chi-square test; # Significance by ANOVA one-way for group factor and Post Hoc tests(Age: EVCO vs. Placebo (*p* = 0.008) and EVCO vs. Placebo-Training (*p* = 0.043); HDL: EVCO vs. EVCO-Training (*p* = 0.013) and EVCO-Training vs. Placebo-Training (*p* = 0.026); LDL: Placebo vs. EVCO-Training (*p* = 0.013); MDA: EVCO vs. EVCO-Training (*p* = 0.042) and EVCO -Training vs. Placebo-Training (*p* = 0.028).
